# Notes on the Treatment of Bronchitis

**Published:** 1896-06-20

**Authors:** Solomon C. Smith

**Affiliations:** Consulting Physician to the Westminster General Infirmary


### Medical Progress and Hospital Clinics.

[*The Editor will be glad to receive offers of co-operation and contributions from members of the profession. All letters should be addressed to* The Editor, at the Office, 428, Strand, London, W.C.]

### NOTES ON THE TREATMENT OF BRONCHITIS.

By Solomon C. Smith, F.R.C.P., Consulting Physician to the Westminster General Infirmary.

(*Continued from page* 174.)

In children it has always to be borne in mind that a common cause of the recurrence of chronic bronchitis, and of the fatality of bronchitis in its various acute forms, is the co-existence of rickets. Wherever this condition exists along with chronic bronchitis it is far better to give cod liver oil and rational diet, together with preparations of iron, than merely to treat the cough, as is so often done, by means of expectorants. In acute cases, unfortunately, there is no time to treat the rickets; nevertheless, the osseous deficiency must be considered in the treatment, deep respiration being encouraged by means of frictions with stimulating liniments, and even by cold affusions, and the position of the child being constantly altered to prevent stagnation in dependent or unexpanding parts, and consequent pulmonary collapse. Whenever rickets is present the mildest case of acute bronchitis should be treated as one of considerable gravity.

Acute capillary bronchitis or broncho-pneumonia is the common cause of death in whooping-cough and measles among the poor, and even among the well-to-do who live amid imperfect sanitary surroundings. It seems almost impossible to draw a dividing line between the finer forms of capillary bronchitis and that condition of broncho-pneumonia which almost certainly arises from the septic effusion in the bronchi being drawn into the alveoli of the lungs. This is not the place to discuss the part played by the various organisms which have been found in such cases, but there seems no doubt that these diseases are usually of a microbic nature. In attempting, then, to remove the cause of the malady it is of the first importance that the patient should be removed from the bad surroundings which have given to the disease its fatal type. In the houses of the well-to-do, whenever bronchitis tends towards the capillary type, the patient should be removed into the most airy room available. Too often it is the case that nurseries occupy positions which are far from sanitary, being in the neighbourhood of housemaids' closets and ventilating into or out of back staircases, and when this is the case it will not uncommonly be found that the transportation of the little patient into a better room will produce an immediate improvement. Among the children of the really poor, whose surroundings are often very bad, the effect of removal to the pure air of a hospital, the emptying of the tubes by an emetic, and the production of deep inspirations by cold affusion, is most striking. Unfortunately, in the cases which are the most fatal, those complicating measles and whooping-cough, such a treatment is but seldom available. Even then, however, it should be recognised that foulness of air is more injurious than cold, and that warmth gained at the expense of ventilation is but a fatal gift of euthanasia.

It is but in accordance with general principles to endeavour to relieve the tubes of as much of their work as possible when they are inflamed. Now, one of their functions is to warm the air, and another is to moisten it, hence it is only reasonable to believe that in cold weather it will be best to ensure that the air is brought artificially to that condition in which it can be breathed with least strain upon the bronchial tubes. This is commonly done by means of a bronchitis kettle which, besides moistening the air, has the great advantage of acting as a useful distributor of heat, watery vapour being a much more effectual agent than dry air in carrying heat to distant parts of the room. But it must not be forgotten that a warm atmosphere, at or near the point of saturation with watery vapour, is depressing and unhealthy, so that care should be taken not to overdo the kettle. Certainly the air should not approach so near to the saturation point as to bring the wet and dry bulb thermometers within two degrees Fahr. of each other, and three degrees would probably be a better limit. According to the Act for regulating the amount of vapour which may be introduced into weaving sheds, there must always be a difference of at least two degrees between the wet and dry bulbs up to a temperature of 70 degrees, a temperature which should never be exceeded in the sick-room, 65 degrees Fahr. being the best temperature to aim at in cases of bronchitis. It must always be remembered that the benefit of the bronchitis kettle is a negative rather than a positive one, and that moisture of air, and even warmth of air, are too dearly purchased if they are to be had only at the expense of freshness.

The third great object in the treatment of bronchitis, viz., the removal of the products of the disease, is that to which treatment by drugs is most commonly directed. There are three principal directions in which aid can be most effectively given for this purpose. The secretion may be modified in character, being either rendered more fluid, so that the adhesive masses of mucus may be lifted off their bed and more easily expectorated, or, when excessive, being diminished in amount and rendered more cohesive, so as to be brought more under the influence of cough. Then the activity of the muscles of the tubes may be stimulated, and also perhaps that of such ciliæ as remain, and finally spasm of the tubes may be relieved.

It is unnecessary here to enumerate the multitude of drugs which have been used for these various purposes. It is desirable, however, to point out that practically the only way by which waste products are removed from the bronchi is by coughing, while, on the other hand, all coughing which does not subserve this purpose is definitely injurious. To promote expectoration, to render cough effective, and at the same time to check all cough which is useless, are the main aims of this side of the treatment of bronchitis.

Now, in regard to this, it is worth bearing in mind that it is impossible to cough efficiently so long as there is spasm of the tubes. Much as the routine use of sedatives in bronchitis is to be deprecated, everyone will have observed cases in which belladonna, stramonium, and even opium, have given an infinity of relief without any apparent injury, even in cases in which from the amount of dyspnœa the administration of any sedative might have seemed to be entirely contraindicated. The fact is that, to a large extent, the beneficial action of sedatives, more especially in acute forms of bronchitis, is due to their influence upon the coincident spasm of the bronchioles, by relaxing which they let in the air and render coughing possible. For this purpose, in addition to the drugs above mentioned, a most useful combination is that of chloral and bromide of sodium. It will often be noticed that when sleep follows the use of such drugs the respiration becomes deeper and slower than before, and when this happens one may be sure that good is being done. When, on the other hand, a sedative produces sleep, but at the same time the respiration continues quick and shallow, the greatest watchfulness must be exercised and sleep must not be allowed to continue for long.

The injurious effects of sedatives arise partly from the action of some of them, such as opium, in "drying up" the bronchial secretion, and thus rendering it more difficult to expectorate, but more especially from their inhibitory effect upon the respiratory centre—a breaking of the reflex arc involved—which leads to the abolition of cough at a time when the tubes are charged, and when by coughing alone they can be cleared. This is the way that soothing syrups kill, but it must not therefore be imagined that any broad rule can be laid down prohibiting the use of sedative medicines in such conditions, for cases occur, in which there is still a fair respiratory capacity, notwithstanding the choked condition of the tubes, but where an ineffectual, useless cough is exhausting the patient, in which the directly sedative effect even of opiates may be used with great advantage as a means of gaining time for the phlegm to "loosen," that is, for its character to change from the less to the more fluid form. This being the object of their administration, such drugs must be given in sufficient dose to have the desired effect, that is, to hold the cough in check for a time, and it is clearly an advantage that they should be combined with such other drugs as are likely to modify the secretion in the direction desired. This, no doubt, is the secret of the success of that time-honoured medicine, "Dover's Powder," which, given in a single efficient dose, is often most effectual in procuring sleep and enabling the patient on awaking to get rid of his accumulated expectoration.

As a means of relieving irritative cough, the inhalation of chloroform is sometimes very useful. Unfortunately, as a result of accidents which have followed upon ill-judged methods of administration, it has fallen somewhat into ill-repute. If, however, it be properly used, it seems to be free from danger.

The best way is to prescribe it as a mixture of one part of chloroform and nine parts of spirits of wine, of which not more than a fluid drachm is to be placed (by the nurse, *not* by the patient) on a very small sponge fixed in a Yeo's inhaler. The chloroform does not evaporate very quickly out of a mixture of this strength, and it is impossible to inhale a dangerous quantity if care be taken that the sponge is of such a size that it will not hold more than the proper dose. It is curious how quickly such an inhalation will in some cases check useless and vexatious cough, and it may be stated generally that if relief be not quickly obtained, it is not desirable to persist with the remedy.

Many means are employed to empty and to purify dilated tubes, among which may be mentioned emetics, stimulating expectorants, various dry inhalations, the use of a spray of ipecacuanha wine, the intratracheal injection of antiseptics, and the use of external applications, such as the Linimentum Terebinthinæ Aceticum. It is not very clear how liniments act in such cases, but that they do good is certain. Probably in children the firm pressure of the hand tends to dislodge inspissated matter from the bronchi—a sort of massage of the lung. It must again be insisted on that to empty the bronchial tubes air must enter freely and must penetrate deeply. Free sponging of the chest with cold water is of very great efficacy. It must be done very quickly, but the water should be cold, and if, in the case of a child, either the rubbing or the sponging sets up a momentary scream so much the better. A child rarely breathes so deeply as when it cries.

A treatise could be written on the drug treatment of bronchitis and its results. This, however, is beyond my present purpose. I would, however, give a word of warning against falling in with the popular error of regarding cough as synonymous with bronchitis, for in a large number of cases of cough there is no bronchitis, and the multitudinous remedies for bronchitis would be quite inappropriate.

